# Proximal aortic repair in asymptomatic patients

**DOI:** 10.1016/j.xjon.2021.05.001

**Published:** 2021-05-13

**Authors:** Emelie Carlestål, Melih Selcuk Ezer, Anders Franco-Cereceda, Christian Olsson

**Affiliations:** a Department of Molecular Medicine and Surgery, The Karolinska Institutet, Stockholm, Sweden; b Department of Cardiothoracic Surgery, Karolinska University Hospital, Stockholm, Sweden

**Keywords:** aorta, elective operation, outcomes, guidelines, ATAAD, acute type A aortic dissection, BAV, bicuspid aortic valve, CRRT, continuous renal-replacement therapy, HCA, hypothermic circulatory arrest, PCS, postcardiotomy shock, VSRR, valve-sparing root replacement

## Abstract

**Objective:**

Current guidelines for elective proximal aortic repair are applicable to elective first-time procedures in asymptomatic patients without other primary indications or connective tissue disorders and with specified aortic diameter or growth rate. The objective was to characterize the surgical outcomes in this narrowly defined patient-population.

**Methods:**

Guideline-compliant patients were identified from a recent (2014-2019) single unit consecutive surgical cohort (n = 935) by excluding total arch replacements, redos, acute and symptomatic patients, and genetic syndromes. Remaining patients were included regardless of surgical procedure performed. Early (30-day or in-hospital) and 1-year mortality were primary outcome measures. Major complications (stroke, severe renal or respiratory insufficiency, postcardiotomy shock, deep sternal wound infection, permanent pacemaker, and re-exploration) up to 1 year postoperatively were secondary outcome measures.

**Results:**

In the resulting study population (n = 262), median age was 63 (interquartile range, 52-71) years, and median surgical risk (European System for Cardiac Operative Risk Evaluation II) was 3.2% (2.0%-4.4%). Early mortality was 2 of 262 (0.76%) without additional deaths up to 1-year postoperatively. The occurrence of major complications was low: stroke, 2 (0.76%); renal insufficiency, 2 (0.76%); respiratory insufficiency, 1 (0.38%); postcardiotomy shock, 1 (0.38%); deep sternal wound infection, 0; permanent pacemaker, 3 (1.1%); and re-exploration, 20 (7.6%), all occurring in the immediate (30-day) postoperative period and without additional events up to 1 year postoperatively.

**Conclusions:**

In this recent cohort including the target population referred to by and managed in accordance with current guidelines, mortality and major complications were exceptionally infrequent. Guidelines should adequately weigh risks of conservative management against current surgical outcomes.

## Graphical abstract

**Figure:**
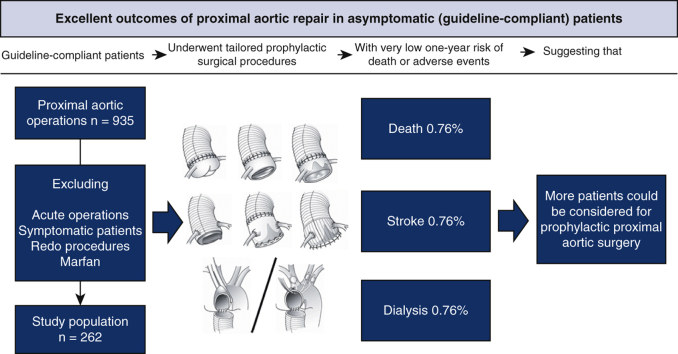

**Figure:**
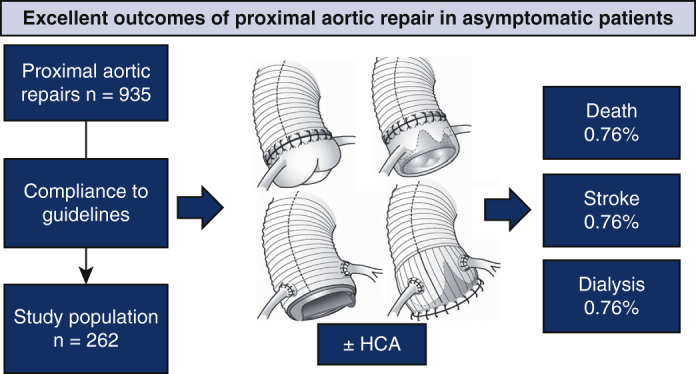
Very few deaths and major complications after prophylactic proximal aortic operations.

**Central Message**

Death and major complications were extremely uncommon up to 1 year after guideline-compliant prophylactic proximal aortic operations in asymptomatic patients.

**Perspective**

The risk of adverse events (ie, death or life-altering complications) after prophylactic proximal aortic surgery according to any valid instance covered by current guidelines was extremely low, regardless what type and extent of surgical procedure was performed. It is essential that guidelines adequately weigh the risk of acute aortic events against current surgical outcomes in the target population.

See Commentary on page 10.

The overall incidence of acute aortic events, mainly acute type A aortic dissection (ATAAD), seems to increase despite more elective proximal aortic operations being performed annually.^1^, ^2^, ^3^ For asymptomatic patients, elective operations aim to prevent acute events: estimated ATAAD case-fatality rate is 73%.^4^ Classically, surgical ATAAD mortality has remained at 18% to 22% in large patient cohorts.^5^^,^^6^ More prophylactic procedures performed could help reduce the ATAAD incidence, provided they are safe and produce favorable outcomes.

Current society-endorsed guidelines (Table E1) largely base the indication for prophylactic proximal aortic operations on the maximal aortic (root or ascending) diameter—5.5 cm in individuals without specific risk factors or 5.0 cm in patients with bicuspid aortic valve (BAV)—and the presence of risk factors such as hypertension, coarctation, or accelerated aortic growth.^7^, ^8^, ^9^ In smaller stature, lower thresholds adjusted to height are proposed.^7^ The thresholds are based on earlier studies on natural history^10^ but have been questioned since a majority of ATAAD occurs at smaller proximal aortic diameters.^11^^,^^12^ In a recent meta-analysis, the annual aortic event rate was 2.2% per patient-year already at a mean proximal aortic diameter of 4.3 cm,^13^ and an expanded analysis of the substantially enlarged Yale population showed a hinge-point of 5.25 cm associated with a steep increase in acute aortic events or death.^14^

The indication for operation also depends on the surgical risk. Therefore, adequate appreciation of surgical risk is instrumental to decision-making. As pointed out earlier,^15^ for patients undergoing prophylactic proximal aortic operations, surgical risk consists not only of death but also of severe, life-altering, complications, affecting longevity or quality of life.

Numerous reports focus on outcomes of specific proximal aortic operations, eg, aortic root replacement, ascending aortic replacement, and hemiarch aortic replacement,^16^, ^17^, ^18^, ^19^, ^20^ but fail to focus on the very patient population referred to by guidelines when quoting the aortic diameter threshold, ie, elective first-time proximal aortic procedures in nonsyndromic asymptomatic patients.^7^, ^8^, ^9^ Patients in this population may need a variety of procedures to address their specific aortic pathology yet are united by their clinical definition and constitute the only population whose outcomes are relevant, in this context, to weigh against natural history risk.

The aim of the present study was to outline contemporary surgical outcomes in prophylactic proximal aortic surgery in the exact patient population referred to by guidelines. Such data are indispensable to evaluate the adequacy and relevance of current guidelines. The primary outcome measure was mortality (death within 365 days of operation). The secondary outcome measures were major complications (within 365 days of operation): stroke, severe renal or respiratory failure, deep sternal wound infection, postcardiotomy shock, permanent pacemaker, or re-exploration and their combination to define an uneventful course in their absence.

## Methods

The study was approved by the Swedish ethical review authority (no 2020-02503 on 8/26/20), with a waiver to obtain written informed consent from individual patients.

### Study Population

**Figure 1:**
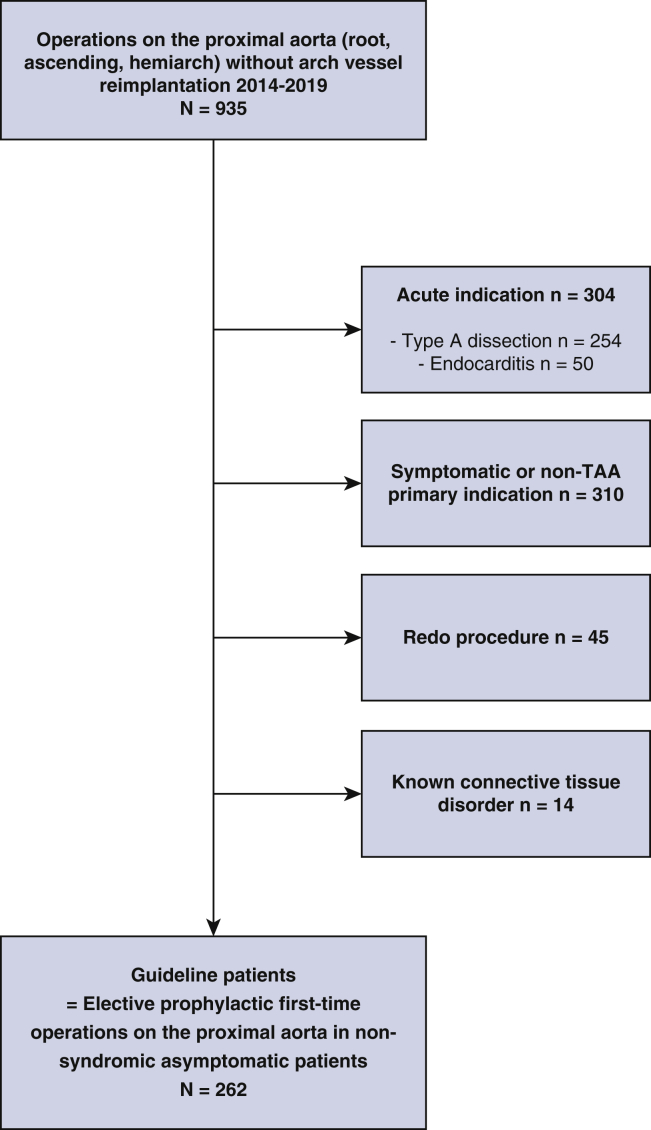
Flowchart of all patients (n = 935) and exclusion criteria applied to define a guideline-compliant study population (n = 262) of asymptomatic patients undergoing prophylactic proximal aortic surgery. *non-TAA*, Non-thoracic aortic aneurysm.

All consecutive patients undergoing procedures on the proximal aorta in a single unit from January 1, 2014, to December 31, 2019, were identified. To obtain a study population unequivocally corresponding to that with a guideline-compliant indication, a set of exclusion criteria were applied: arch replacement, acute procedures, procedures for symptomatic conditions or with other primary indication, redo procedures, and, finally, Marfan syndrome or other known connective tissue disorders (Figure 1). Any of the exclusion criteria would have either prompted an operation and therefore constituted the primary indication for operation, or modified the decision on when to operate, rendering the diameter-related or growth rate–related guideline criteria inapplicable. The proportion of patients undergoing aortic valve procedures (replacement or repair) were essentially asymptomatic (less than New York Heart Association class II), ie, without stand-alone indication for valve surgery. Included patients with aortic insufficiency did not have objective (by echocardiography or magnetic resonance imaging) signs of left ventricular dysfunction. Included patients with aortic stenosis had transvalvular peak velocities <3 m/s. In effect, the study population had proximal aortic dilatation as the sole indication for operation at the point in time when the operation was performed. Patients with bicuspid aortic valve were included, provided they too met their specific aortic diameter criteria and were asymptomatic.

**Figure 2:**
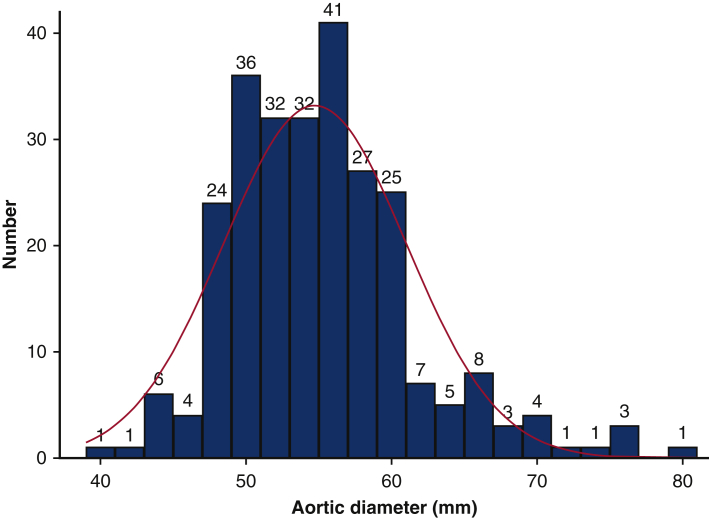
Distribution of aortic diameter (millimeters) at time of proximal aortic repair in asymptomatic patients. Number of patients in each 2-mm interval on top of bars. *Red curve*, approximated normal distribution.

Data from medical records were retrospectively collected. Patients were grouped according to main type of proximal aortic procedure: supracoronary graft alone (or with aortic valve repair only), supracoronary graft with separate aortic valve replacement, composite (biological or mechanical) root replacement, and valve-sparing root replacement, respectively, and further according to mode of distal anastomosis: with aortic crossclamp or open during circulatory arrest (Figure 2).

### Variable Definitions

Definitions of outcome measures (death and major complications) were in accordance with recently published standards of reporting.^21^ To match the generally employed annual aortic event risk, all outcomes occurring within 365 days of the index operation were included. Death was also reported as early (30-day or in-hospital) mortality and on follow-up. Stroke was defined as new disabling symptoms not completely resolving before discharge and verified with radiological examination. Severe renal failure was defined as new-onset unplanned need for continuous renal-replacement therapy (CRRT). Severe respiratory failure was defined as need for tracheostomy, as indicated by prolonged (>14 days) period of mechanical ventilation and/or repeated failure to wean off ventilator. Postcardiotomy shock (PCS) was defined as heart failure with need of continuous inotropic (ie, not only vasopressor) or mechanical circulatory support. Deep sternal wound infection was defined as culture-positive wound infection with sternal involvement and surgical reintervention during index hospitalization. Re-exploration for bleeding or tamponade included any surgical re-exploration made on clinical suspicion. Permanent pacemaker implantations were performed to treat new high-degree atrioventricular block. The European System for Cardiac Operative Risk Evaluation II was used to estimate surgical risk. Aortic diameter was obtained from the most recent computed tomography scan preceding surgery.

### Surgical Procedures

With few exceptions, a standard median sternotomy was performed and central (aortic and atrial 2-stage) cannulation used for cardiopulmonary bypass. The left ventricle was vented and wound cavity carbon dioxide insufflation used to reduce risk of air embolization. For myocardial protection, cold (4°C) blood cardioplegia was delivered intermittently (15-30 minutes) ante- and retrogradely. Normothermia was employed for cases operated on aortic crossclamp. For hypothermic circulatory arrest (HCA), moderate (28°C-30°C bladder temperature) hypothermia was used. After opening the aorta and exsanguinating the patient, 3 separate cerebral perfusion cannulae were introduced into the cervical vessel orifices and cold (20°C) selective antegrade cerebral perfusion maintained at 5-600 mL/min, with near-infrared spectroscopy (INVOS 5100C; Medtronic, Boulder, Colo) monitoring of regional cerebral oxygenation.

The surgical repair always included ascending aortic graft replacement. The aortic valve was repaired or replaced with either a mechanical or a biological valve prothesis when judged appropriate and according to patient preference. Valve repair techniques included cusp plication, cusp suturing, and subannular ring plasty. If dilated, the aortic root was replaced using a composite (mechanical or biological) graft or as valve-sparing root replacement (VSRR) using the reimplantation technique within a sinus-shaped aortic root vascular prosthesis (Gelweave Valsalva; Vascutek, Renfrewshire, Scotland). The coronary ostia were reimplanted as free buttons. Distally, replacement was extended to resect pathologically dilated aorta. In general, open distal anastomosis was preferred if ascending aortic diameter remained dilatated to 40 to 45 mm at projected site of distal anastomosis. Other concomitant cardiac procedures were performed as indicated.

### Statistical Methods

Data were reported as medians with interquartile ranges or numbers (n) with percentages and/or ranges. The study was not hypothesis-testing and data were descriptive only. Survival was estimated using Kaplan-Meier methods. Data were procured using Stata, version 16, software (Stata Corp, College Station, Tex).

## Results

**Figure 3:**
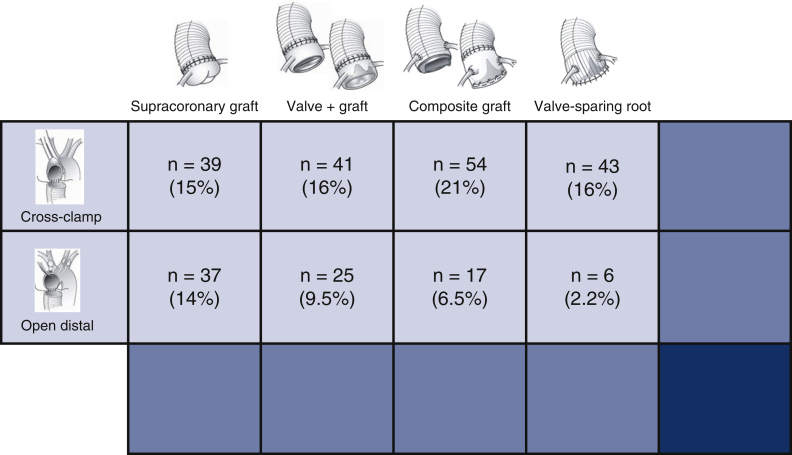
Number of patients undergoing each type of proximal aortic surgical procedure (supracoronary graft; supracoronary graft and valve replacement; composite aortic root replacement; and valve-sparing root replacement, respectively) with aortic crossclamping or open distal anastomosis. Each category is summarized and each percentage related to the overall study population (n = 262).

During the study period, 935 proximal aortic operations were performed. After the application of exclusion criteria, the remaining 262 (42% of nonacute) asymptomatic patients undergoing elective prophylactic operations on the proximal aorta formed the study population (Figure 1). Median age was 60 years, and 70% were men (Table 1). Aortic diameter ranged from 40 to 80 mm with a median of 55 mm (Figure 2). Composite aortic root replacement without HCA was the single most common surgical procedure, whereas supracoronary graft replacements were most common when including HCA (Figure 3). Overall, 170 patients (65%) had simultaneous aortic valve procedures: 33 (19%) repair and 137 (81%) replacement. Valve repair was performed in combination with a supracoronary graft in 16 (48%) and in combination with VSRR in 17 (52%). For aortic valve replacement, biological prostheses were used in 90 (66%) and mechanical in the remaining 47 (34%). Valve replacement was indicated primarily for aortic stenosis in 6 patients (4.4% of valve replacements), regurgitation in 97 (71%), and combined stenosis and regurgitation in the remaining 34 (25%). Patients receiving a mechanical aortic valve prosthesis had a lower median age, 51 (43-57) years, than those receiving a biological prosthesis, ie, 70 (65-74) years. BAV was present in 105 of 262 (40%) patients with a median age of 54 (44-63) years. Patients with BAV more often had aortic valve procedures: 86 of 105 (82%) versus 84 of 157 (54%) in non-BAV and median aortic diameter was 52 mm compared with 56 mm with tricuspid aortic valve.

**Table 1:** Overall clinical, surgical, and perfusion characteristics of asymptomatic patients undergoing first-time elective proximal aortic operation (n = 262)

Variable	n (%) or median (IQR)	Range
Clinical characteristics		
Sex, male	184 (70)	
Age, y	63 (52-71)	20-85
Height, cm	178 (170-184)	149-199
Weight, kg	82 (70-93)	54-153
Body mass index, kg/m^2^	26 (23-29)	19-47
Hypertension	155 (59)	
Diabetes	10 (3.8)	
Bicuspid aortic valve	105 (40)	
Family history of aortic disease	31 (12)	
Left ventricular ejection fraction, %	60 (55-60)	30-70
EuroSCORE II, %	3.2 (2.0-4.4)	1.0-18.3
Maximal aortic diameter, mm	55 (50-58)	40-80
Surgical procedures		
Supracoronary graft	76 (29)	
With aortic valve repair	16 (6.1)	
Supracoronary graft + valve replacement	66 (25)	
Mechanical prostheses	16 (6.1)	
Biological prostheses	50 (19)	
Composite graft	71 (27)	
Mechanical composite	31 (12)	
Biological composite	40 (15)	
Valve-sparing root	49 (19)	
With aortic valve repair	17 (6.5)	
Perfusion characteristics		
With aortic crossclamp	177 (68)	
Cardiopulmonary bypass, min	128 (100-159)	38-316
Aortic crossclamp, min	100 (70-130)	15-296
With open distal anastomosis	85 (32)	
Cardiopulmonary bypass, min	141 (121-173)	87-316
Hypothermic circulatory arrest, min	23 (20-28)	14-48
Antegrade cerebral perfusion, min	15 (12-20)	8-43

*IQR*, Interquartile range; *EuroSCORE*, European System for Cardiac Operative Risk Evaluation.

Of the patients who underwent valve repair, none had more than mild (grade I/IV) aortic regurgitation at discharge and none was reoperated for repair failure during the 1-year observation period. In 1 patient, a VSRR was intraoperatively converted to mechanical aortic valve replacement due to unacceptable degree of aortic regurgitation and grouped as composite graft replacement, yielding a primary 1 of 50 (2%) VSRR failure rate. A total of 32 concomitant procedures were performed in 28 (11%) patients: 16 coronary bypass, 7 atrial septal defect closures, 5 procedures for atrial fibrillation, and 4 mitral valve procedures.

Two male patients (0.76%) died early, for an observed rate/expected rate ratio (based on surgical risk according to European System for Cardiac Operative Risk Evaluation II) of 0.24. One (biological composite on crossclamp) had an uneventful postoperative recovery but suffered lethal hemorrhage after chest tube placement to drain pleural effusion. The other (biological composite with open distal anastomosis) postoperatively developed acute occlusion of the left main stem, underwent emergency off-pump vein graft bypass to LAD, developed PCS, and died after 10 days on mechanical circulatory support without myocardial recovery. This patient also accounted for 1 of 2 strokes and the only tracheostomy. The other stroke occurred in a 72-year-old woman, operated with a supracoronary graft and hemiarch repair in HCA, with new right-sided hemiparesis postoperatively. Three permanent pacemakers were implanted postoperatively, all due to high-degree atrioventricular block and all in patients, aged 51, 70, and 75 years, respectively, undergoing root (2) and/or valve replacement (1) procedures. Two patients required CRRT for new-onset renal failure. In both, CRRT was discontinued before discharge, and serum creatinine levels were completely normalized.

**Figure 4:**
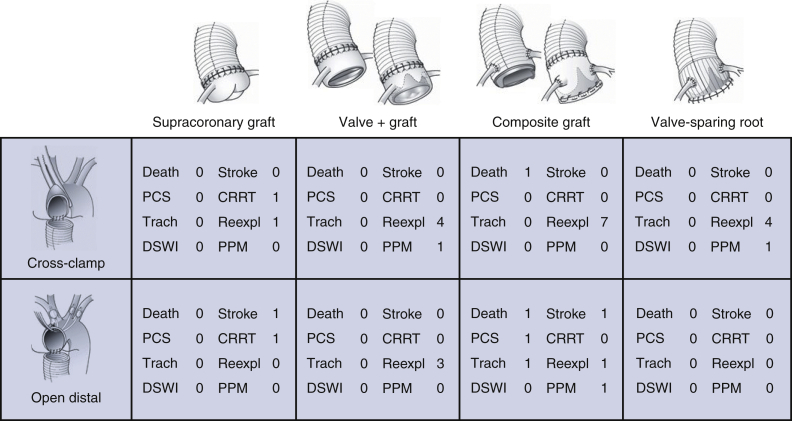
Number of each major adverse event occurring at up to 1-year follow-up for each type of proximal aortic surgical procedure (supracoronary graft; supracoronary graft and valve replacement; composite aortic root replacement; and valve-sparing root replacement, respectively) with aortic crossclamping or open distal anastomosis. *PCS*, Postcardiotomy shock; *CRRT*, continuous renal-replacement therapy; *Trach*, tracheostomy; *Reexpl*; re-exploration for bleeding/tamponade; *DSWI*, deep sternal wound infection; *PPM*, permanent pacemaker.

**Figure 5:**
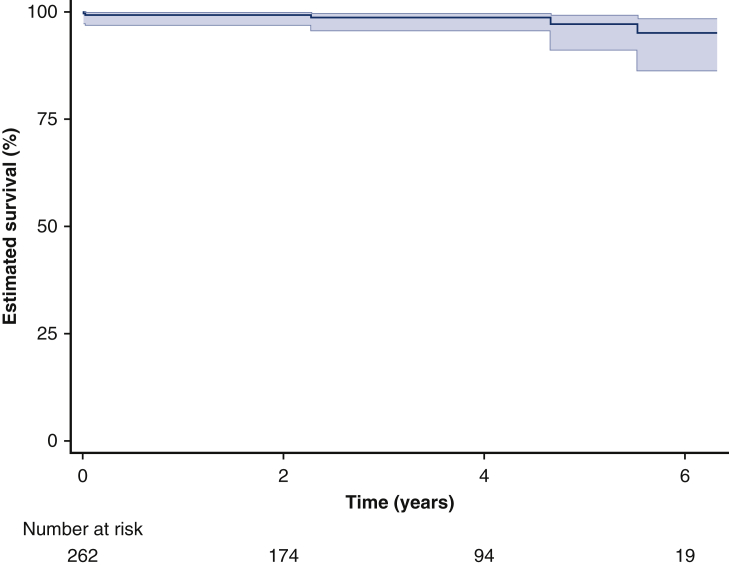
Estimated (Kaplan–Meier) survival with 95% confidence intervals in asymptomatic patients undergoing elective prophylactic surgical repair of the proximal aorta. Estimated 5-year survival was 97% (95% confidence limits, 91%-99%).

The most common major complication was bleeding/tamponade requiring re-exploration in 20 (7.6%) patients; 15 of 20 (75%) in patients undergoing valve-replacement procedure (with or without root replacement). None of these had other secondary major complications. No patient developed deep sternal wound infection. Overall, 234 (89%) patients had an uneventful postoperative course, 253 (97%) if deducting re-explorations. Within 365 days postoperatively, no additional deaths or any of the major complications occurred (Figure 4). Follow-up regarding vital status and major complications was 100% complete. During 829 patient-years cumulative follow-up (median 2.9, max 6.3 years), there were 5 additional deaths. Two were nonaortic (lung cancer and coronavirus disease 2019, respectively). One was a confirmed aortic arch rupture, and 2 unknown were classified as possible aortic events, for a probability of >95% freedom of aortic-related death at 5 years postoperatively (Figure 5), corresponding to a linearized risk of 0.6 deaths/100 patient-years. Time to death was 2.3-5.5 years and patients were 71, 80, and 81 years old at time of operation.

## Discussion

**Figure 6:**
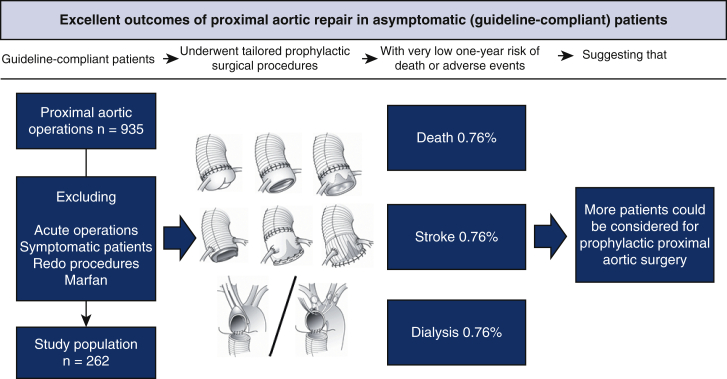
Guideline-compliant patients (n = 262) underwent individualized surgical treatment with excellent 1-year outcomes. *HCA*, Hypothermic circulatory arrest.

Summarized in Figure 6, the outcomes in this guideline-compliant contemporary cohort of 262 consecutive prophylactic proximal aortic operations were quite satisfactory. With 1-year postoperative observation time, overall mortality remained at initial 0.8%, as did other major complications in the 0% to 1.5% range. The overall prevalence of uneventful course was 97%, save re-exploration, which occurred in 7.6% but did not entail further complicated postoperative course in any patient. All procedures were tailored to meet individual needs of extent and outcomes were similar regardless of procedure type, regardless if HCA was employed or not and regardless of aortic valve morphology and procedures. The rate of possible aortic mortality (0.6/100 patient-years) was low and affected elderly patients. Concerns for medium-term survival, especially in younger patients, should not deter from prophylactic operation.

The target population was preoperatively asymptomatic. Major complications may considerably impact both prognosis and quality of life. Therefore, it was vital to include all such events in the weighing of surgical against nonsurgical risks. Yamabe and colleagues^15^ reported such uneventful recovery in 78% of 676 patients undergoing elective aortic root replacement, as compared with 89% in the present study. Their outcomes, however, were not applicable to the prophylactic patient profile since both redo procedures and symptomatic patients were included. Wallen and colleagues^22^ reported recent encouraging STS database data (n = 8807) on elective aortic root replacement: 2.2% early mortality, 1.4% early stroke, and overall 82% uneventful recovery (without reporting PCS or permanent pacemaker implantation).

Contemporary single-center outcomes of proximal aortic operations are excellent. Early mortality 0% to 1.1%,^16^, ^17^, ^18^ early stroke 0%,^18^ and major complications (renal and respiratory failure) <10% even for hemiarch replacement.^19^^,^^20^ However, these and many similar studies had in common a lack of 1-year observations on all end points and the featuring of one or few specific surgical procedure(s) rather than one well-outlined patient population: those with an exclusively prophylactic surgical indication.

The current guideline aortic diameter cutoffs seem largely based on the Yale Aortic Institute cohort. Their most recent analysis counted 3400 patients (compared with the earlier 230 forming the basis for the current guidelines) and identified 5.25 cm, rather than earlier 5.9 cm, as the first hinge-point of annual aortic event risk, increasing 5-fold from around 1% to 5%.^14^ Simultaneously, their surgical outcomes improved substantially, now ranging from 0% to 1.9% surgical mortality and 1.0% to 1.4% stroke, respectively, as compared with 4.3% and 8%, respectively, in the earlier era.^10^ Indeed, 2011 to 2017, the Yale group applied 5.0-cm aortic diameter (or 4.0-4.9 cm in presence of selected risk factors: symptoms, family history, connective tissue disease, or BAV) as size criterion for prophylactic surgery, with resulting 1% (5/472) hospital mortality.^23^

There are no studies dedicated to watchful waiting in 50- to 55-mm proximal aortic aneurysms. A study including 186 patients with 46- to 50-mm root/ascending aneurysms found only 1 (1/186, 0.54%) acute event, but another 22 underwent prophylactic thoracic aortic surgery for a linearized rate of approximately 2.8% per year of acute event or operation.^24^ Another study in even smaller (mean 4.4 cm) aneurysms found no acute events, and 3 of 232 (1.29%) underwent prophylactic operation.^25^ Surgical mortality was zero in both series. Acute aortic events seemed uncommon, but notably so in conjunction with a substantial rate of pre-emptive operations. Ideally, a multicenter trial randomizing patients to elective proximal aortic repair versus watchful waiting would be needed to firmly establish the role of prophylactic surgery and provide higher-level evidence. One such study is currently registered and recruiting (TITAN:SvS [Treatment in Thoracic Aortic Aneurysm: Surgery vs Surveillance], clinicaltrials.gov identifier NCT03536312).

The present study was undertaken in a high-volume equivalent unit, yet with a limited annual overall caseload (n ≈ 1000). No patient was referred from other cardiothoracic units. Patients were consecutive and unselected apart from criteria applied to define the study population. All surgical procedures were in common use and performed by a number of surgeons with variable surgical volumes and experience. These aspects are important for the generalizability of the results, which in turn is paramount to the discussion of adjusting universally applied treatment guidelines.^26^ Reduced prevalence of acute aortic events and reduced mortality of thoracic aortic disease cannot be inferred from lowered aortic diameter thresholds for elective operations. However, when current surgical practice, applied to the appropriate patients and evaluated comprehensively in year-long perspective, produce outcomes that supersedes those of watchful waiting, it remains sound to consider lowered thresholds as a means to offer effective treatment to a larger population and possibly prevent more acute events.

### Study Limitations

This study has several important limitations. In the interest of creating a truly contemporaneous study, the study period was not stretched further than 2014, and hence the study population size is limited and long-term outcomes not yet available. There were overall too few outcomes to allow meaningful statistical analysis and groupwise comparisons; hence, data are descriptive only. In effect, the safety of applying a lower surgical threshold could not be demonstrated in this descriptive study setting.

### Conflict of Interest Statement

The authors reported no conflicts of interest.

The *Journal* policy requires editors and reviewers to disclose conflicts of interest and to decline handling or reviewing manuscripts for which they may have a conflict of interest. The editors and reviewers of this article have no conflicts of interest.

## Figures and Tables

**Table E1:** Summary of current guideline aortic diameter thresholds for surgical indication, for the aortic root and ascending aorta with bicuspid and tricuspid aortic valve, respectively

	ACCF et al 2010	CCS 2014	ESC 2014
Aortic root TAV	55 mm	55 mm∗	55 mm†
Ascending TAV	55 mm	55 mm∗	55 mm†
Aortic root BAV	πr^2^/h > 10‡	50-55 mm∗	55 mm§
Ascending BAV	πr^2^/h > 10‡	50-55 mm∗	55 mm§
Quoted surgical risk	2.5%-5.0%‖	1%¶	n/a
Quoted annual aortic event risk	14%#	7%-12%	14%#

Surgical and annual acute aortic event risk quoted by each guideline. *ACCF*, American College of Cardiology Foundation; *CCS*, Canadian Cardiovascular Society; *ESC*, European Society of Cardiology; *TAV*, tricuspid aortic valve; *BAV*, bicuspid aortic valve.

∗ “Size thresholds for intervention should take body patient size into consideration, either empirically or using proposed formulas for adjustment.”

† “Lower thresholds for intervention may be considered according to body surface area […] in the case of rapid progression, aortic valve regurgitation, planned pregnancy, and patient's preference.”

‡ For a normal-height man (1.82 m), corresponding to aortic diameter ≈ 48 mm. For a normal-height woman (1.67 m), aortic diameter ≈ 46 mm.

§ 50 mm if additional risk factor(s): family history, systemic hypertension, coarctation, or increase in aortic diameter >3 mm/year.

‖ References (n = 4) from 1997 to 2002. Also expressed in text as “less than 5%.”

¶ No reference provided.

# At aortic diameter “hinge-point” 6.0 cm.
